# Running Performance Decline in IRONMAN-Distance Triathlon: A Scoping Review of Existing Evidence and Research Gaps

**DOI:** 10.3390/sports14080345

**Published:** 2026-08-10

**Authors:** Yujiro Tsutsumi, Shuichi Machida, Hisashi Naito

**Affiliations:** Graduate School of Health and Sports Science, Juntendo University, 1-1 Hirakagakuendai, Inzai, Chiba 270-1695, Japan; machidas@juntendo.ac.jp

**Keywords:** pacing strategy, marathon, age-group athletes, bike-to-run transition, running economy, running kinematics, inter-individual variability, between-context decline

## Abstract

In an IRONMAN-distance triathlon, run performance is a particularly strong correlate of overall race outcome, alongside cycling. Although individual research areas such as pacing, performance prediction, and laboratory physiology have been reviewed separately, they have not been integrated to determine factors contributing to running performance decline or to identify key knowledge gaps. This scoping review aims to map the current evidence on running performance decline in IRONMAN-distance triathlon and identify key gaps, with a particular focus on age-group athletes. Following the Arksey and O’Malley framework and PRISMA-ScR guidelines, we searched PubMed and SPORTDiscus for studies reporting quantitative running performance outcomes in IRONMAN races or bike-to-run transition protocols. Of 896 deduplicated records, 70 studies were included. Findings were organised into five sub-questions addressing the extent of evidence, quantification of decline, physiological and biomechanical changes, underlying mechanisms, and inter-individual variability. Pacing patterns and performance predictors are well described, whereas mechanistic research relies almost entirely on short, controlled protocols. Critically, no study identified by this review quantified the “between-context decline,” defined as the difference between an athlete’s standalone marathon performance and their actual IRONMAN running performance. Addressing these gaps requires observational studies using real IRONMAN race data and individual-level analyses, particularly for age-group athletes.

## 1. Introduction

In IRONMAN-distance triathlon—consisting of a 3.8 km swim, 180 km bike, and 42.2 km run—the final marathon leg is a particularly strong correlate of overall race outcome, alongside cycling. Large-scale studies have shown that run split time is strongly correlated with overall finish time, to a degree comparable to cycling and markedly greater than swimming [1,2]. Because the run split forms part of the total race time and also reflects fatigue accumulated earlier in the race, the strength of this association does not by itself establish that the run determines the outcome. Age-related performance decline in IRONMAN triathlon is greater in running and swimming than in cycling [3]. Marathon personal best (PB) is consistently among the strongest predictors of IRONMAN race time, along with training volume and Olympic-distance PB [4,5]. However, the substantial unexplained variance in these prediction models implies that differences between faster and slower finishers are determined not only by baseline running ability, but also by the extent to which that running ability is impaired during the IRONMAN race. Overall, performance depends not only on an athlete’s running speed but also on the magnitude of performance decline following the preceding swimming and cycling segment.

Numerous studies have investigated running performance decline in IRONMAN-distance triathlon across several areas. Experimental laboratory studies have measured the effects of cycling on subsequent running, including cardiorespiratory responses, neuromuscular fatigue, stride mechanics, and perceived exertion [6]. On the observational side, researchers have described pacing patterns according to performance group, sex, and age. Separately, prediction studies have identified training variables and prior race times as important correlates of overall IRONMAN performance [1].

These areas have generated valuable findings, but have not directly addressed the central question of this review. Mechanistic studies have shown the physiological impact of the bike-to-run transition under controlled conditions. Observational studies have described population-level race patterns. Prediction studies reveal the factors that correlate with finishing time. However, a critical gap remains: none of these studies have quantified how far an athlete’s IRONMAN running performance differs from their standalone running ability, nor have they explained why this difference varies substantially between individuals.

Running performance decline can be measured in more than one way, and the different measures answer different questions. Within a race, it can be described from the change in running speed over the course of the run. In experimental studies, it is measured as the difference between running after a cycling bout and running without prior cycling; because both runs are performed by the same athletes under matched conditions, this difference can reasonably be attributed to the preceding cycling. A third comparison is between an athlete’s standalone marathon performance and their IRONMAN running performance, which we refer to here as the “between-context decline”. Here the two performances are separated in time and take place on different courses and under different conditions, so this quantity describes the overall difference between two performance contexts rather than the effect of the preceding swim and bike alone: course profile, environmental conditions, equipment, race strategy, and changes in fitness over time all contribute to it. It is also the comparison that is almost absent from the literature, for reasons that include the difficulty of obtaining a comparable standalone reference performance, the impossibility of replicating a full IRONMAN race in the laboratory, and the many uncontrolled variables present in real-race data. These difficulties are considered further in Section 4.2.

Given this situation, a systematic mapping of what is known and what is not known is required. Knechtle et al. [1] provided a narrative review of performance predictors, and Rico Bini et al. [6] systematically reviewed bike-to-run transition studies. These reviews cover individual research areas, but the evidence has not been synthesised across those areas to identify the existing knowledge gaps and determine the types of studies needed to address them. The present review is therefore an evidence-mapping exercise: it charts what has been studied, identifies where evidence is absent, and sets out priorities for future research rather than testing a hypothesis.

Age-group athletes are a primary focus of this review for two reasons. First, elite and professional triathletes tend to show smaller running performance declines and less variation among individuals. Second, and more practically, age-group athletes account for the overwhelming majority of IRONMAN finishers. Their training backgrounds, racing experiences, and physiological capacities are much more diverse, resulting in greater inter-individual variability and thereby increasing the potential to identify factors with practical implications for coaching and athlete preparation.

This scoping review addresses the following primary research question: What is the nature and extent of existing research on running performance decline in IRONMAN-distance triathlon, and what key evidence gaps remain, particularly regarding age-group athletes?

In addition, the review addresses the following five sub-questions:

1. To what extent has running performance decline been studied at IRONMAN race distance, as opposed to shorter races and controlled protocols?
2. To what extent has the magnitude of running performance decline been quantified, both within-race and relative to standalone running ability?
3. What physiological and biomechanical changes have been observed during the bike-to-run transition and within the run segment?
4. What causal mechanisms have been identified to explain running performance decline, and how consistent is the evidence?
5. What factors have been identified as contributing to differences in the magnitude of the performance decline across individuals?

## 2. Methods

### 2.1. Methodological Framework

We conducted this scoping review following the framework of Arksey and O’Malley [7] and incorporated methodological enhancements from Levac et al. [8]. Reporting adhered to the PRISMA Extension for Scoping Reviews (PRISMA-ScR) checklist [9]. A formal review protocol was not pre-registered in a public registry; however, the methodological framework, eligibility criteria, search strategy, and data charting fields were defined prior to full-text screening and data extraction.

### 2.2. Eligibility Criteria

Population: Athletes competing in triathlon events, with primary focus on IRONMAN-distance formats (swim: 3.8 km, bike: 180 km, and run: 42.2 km) and age-group (non-professional) participants.

Concept: Studies examining quantitative running performance outcomes in triathlon, including run split times, run speed or pace, within-race pacing patterns, or run-related dependent variables such as stride parameters, distance covered during the run segment, or running performance decline.

Context: Race-data observational studies, field measurement studies conducted during competition, and controlled bike-to-run studies conducted either in the field or in the laboratory were all eligible. The IRONMAN full-distance requirement described below applies only to race-data observational studies.

Inclusion criteria:

• Peer-reviewed original research articles (no language restrictions were applied).
• Studies reporting running performance as an outcome variable; run segment time, speed, pace, distance, or run-related dependent measures (e.g., stride length, pacing profile, and performance decline) had to be quantitatively reported.
• Eligible studies fell into one of four categories, and the distance requirement applied only to the first:
  (i) Race-data observational studies, which analysed race results from official race databases. These had to use IRONMAN full-distance race data (swim 3.8 km, bike 180 km, run 42.2 km).
  (ii) Field measurement studies, which took direct measurements of running performance or running movement patterns during competition. These had to measure running performance decline (decrements in speed or pace over the course of the run) or changes in running movement patterns (stride and cadence parameters, or running kinematics) following a cycling segment. No restriction was placed on race format, because these studies measure the bike-to-run transition directly rather than inferring it from race results.
  (iii) Controlled bike-to-run studies conducted in the field, in which the run was performed overground on a track or on a road course.
  (iv) Controlled bike-to-run studies conducted in the laboratory, in which the run was performed on a treadmill or within a laboratory measurement area.

In both (iii) and (iv), a prescribed cycling bout was followed by a run under conditions set by the investigators. These studies had to measure running performance decline (decrements in speed, pace, time to exhaustion, or distance covered) or changes in running movement patterns (stride and cadence parameters, running kinematics, or muscle activation) following a cycling segment.

Exclusion criteria are as follows:

• Review articles.
• Conference abstracts without full-text availability, unless no full peer-reviewed version was available.
• Studies exclusively examining swim and/or cycle performance without run outcome measures.
• Studies limited to duathlon or aquathlon formats.
• Race-data observational studies that used race results from formats other than the IRONMAN full distance (including middle-distance, Olympic-distance, sprint, and ultra-triathlon events).
• Experimental studies evaluating only physiological cost variables (oxygen uptake [$\dot{V}$O_2_], running economy, heart rate) at a fixed speed without measuring running performance deterioration or movement pattern changes
• Studies for which full text could not be obtained
• Studies addressing related but distinct topics (e.g., predictors of overall race time, demographic correlates, and population-level performance comparisons) in which running performance was not among the outcomes the study was designed to explain.

Studies that did not meet the eligibility criteria were not charted. A subset of such studies is cited elsewhere in this review to situate the findings within the broader IRONMAN literature; these citations are identified as such in the text and do not form part of the charted evidence base.

### 2.3. Search Strategy

PubMed and SPORTDiscus were selected as the primary databases for this review. PubMed indexes the biomedical literature, including exercise physiology, while SPORTDiscus is the principal bibliographic database for sport and exercise science and covers journals that are not indexed in PubMed. Together they were judged to cover the two bodies of literature relevant to this review: IRONMAN race analyses and bike-to-run transition studies.

PubMed was searched via the NCBI interface and SPORTDiscus via EBSCOhost on 25 February 2026. The strategy used a two-block OR structure designed to capture: (a) running performance outcomes in IRONMAN and IRONMAN-distance triathlon (observational block), and (b) bike-to-run transition studies reporting running performance measures (experimental block).

Block 1 combined IRONMAN-distance terminology (e.g., ironman, “IRONMAN-distance triathlon”, and “ultra-distance triathlon”) with running performance terms (e.g., running, marathon, pacing, “run split”, and “race time”). Block 2 combined triathlon population terms (triathlete* and triathlon) with cycling terms (e.g., cycling and bike) and running outcome terms (e.g., running, stride, and “transition run”). Both blocks were merged with OR. All terms were searched within the title and abstract fields and adapted to database-specific syntax. In SPORTDiscus, we used the step-by-step Search History function to avoid bracket misinterpretation, while limiting results to Academic Journals and excluding magazine content. PubMed returned 681 records and SPORTDiscus 600, producing 896 unique records after duplicate removal. The complete search strings for each database are shown in Supplementary Table S2.

### 2.4. Source Selection

Titles and abstracts were screened by the primary author (Y.T.) using ASReview LAB v2.2 (ASReview Project, Utrecht University, Utrecht, The Netherlands) in oracle mode. This tool applies active learning to rank unscreened records by their estimated relevance and presents them to the reviewer in that order.

The model was initialised with 47 records that were labelled before active-learning screening began. These records were identified by searching the imported dataset rather than by random selection: 19 records addressing bike-to-run transition, triathlon performance trends, or IRONMAN pacing, and 28 triathlon-related records that clearly did not meet the eligibility criteria. Both classes were provided so that the classifier had labelled examples of relevant and irrelevant records at the outset. Records were represented using term frequency–inverse document frequency features (unigrams and bigrams, sublinear term frequency), classified with a support vector machine, and balanced during training; the record with the highest estimated relevance was presented at each step. Screening stopped according to a rule specified before screening began: screening ended once 50 consecutive records had been labelled as not relevant.

Screening was completed on 25 February 2026. A further 188 records were screened through active learning (63 relevant, 125 not relevant) before the stopping rule was reached. In total, 237 of the 896 records (26.5%) were labelled by the reviewer, of which 84 were labelled relevant and 153 not relevant. The remaining 659 records (73.5%) were ranked below the stopping point and were not screened. No labelling decisions were subsequently reversed.

After screening stopped, the labelled records were checked against the set of studies known to the review team before the search. This check identified one eligible study that had not been presented during active-learning screening; its records were located in the dataset, screened manually, and labelled relevant. The 84 relevant records corresponded to 81 unique studies: three studies were each represented by two records that had not been recognised as duplicates during deduplication, because their bibliographic details differed slightly between the two databases. These 81 studies were assessed at full text against the inclusion and exclusion criteria.

### 2.5. Data Charting

The data were extracted using the charting approach described by Arksey and O’Malley [7]. For each study, we recorded bibliographic details, participant and protocol characteristics, sub-question tags (1–5), and a summary of the findings and any identified research gaps. Data extraction was conducted by a single reviewer (Y.T.). For studies that also appeared in prior systematic reviews of related topics [6], published data extractions were consulted as a cross-check.

## 3. Results

### 3.1. Study Selection Process

**Figure 1:**
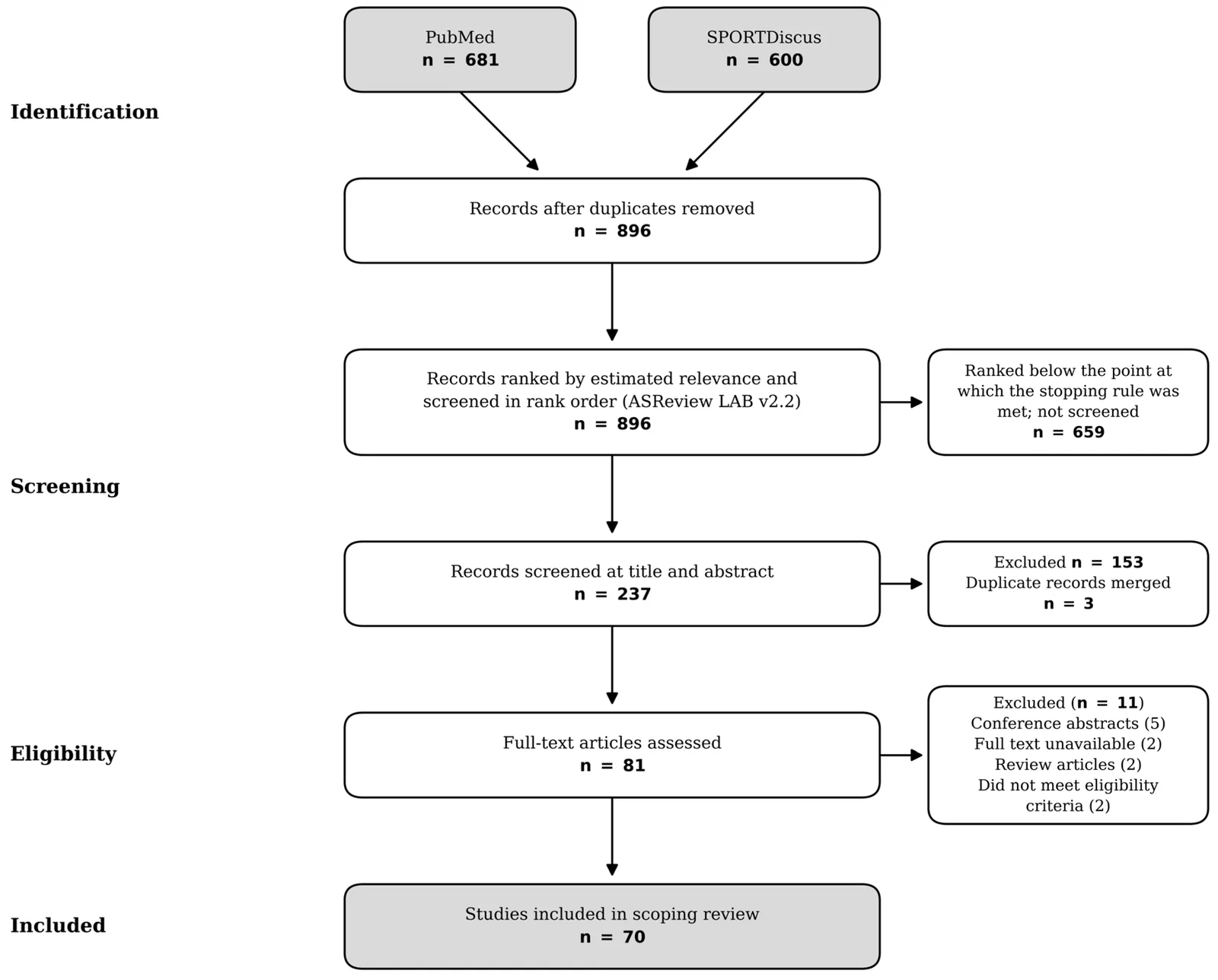
PRISMA-ScR flow diagram representing the study selection process for this scoping review.

The PRISMA-ScR flow diagram summarises the study selection process (Figure 1). Of the 896 deduplicated records, 81 papers underwent full-text review. Eleven studies were excluded from the review: five conference abstracts superseded by full peer-reviewed publications of the same research, two papers whose full texts could not be obtained, two review articles with no original data, and two papers whose primary outcomes did not meet our inclusion criteria. The excluded studies, the reason for each exclusion, and, where it could be established, the full publication that superseded an excluded abstract are listed in Supplementary Table S3. The remaining 70 studies were included in the final analysis.

Studies that used IRONMAN race records to identify predictors of the overall finish time, rather than to analyse running performance itself, were not eligible and were excluded at title and abstract screening; a subset of these is cited in the Discussion to situate the present findings.

### 3.2. Overview of the Included Studies

**Figure 2:**
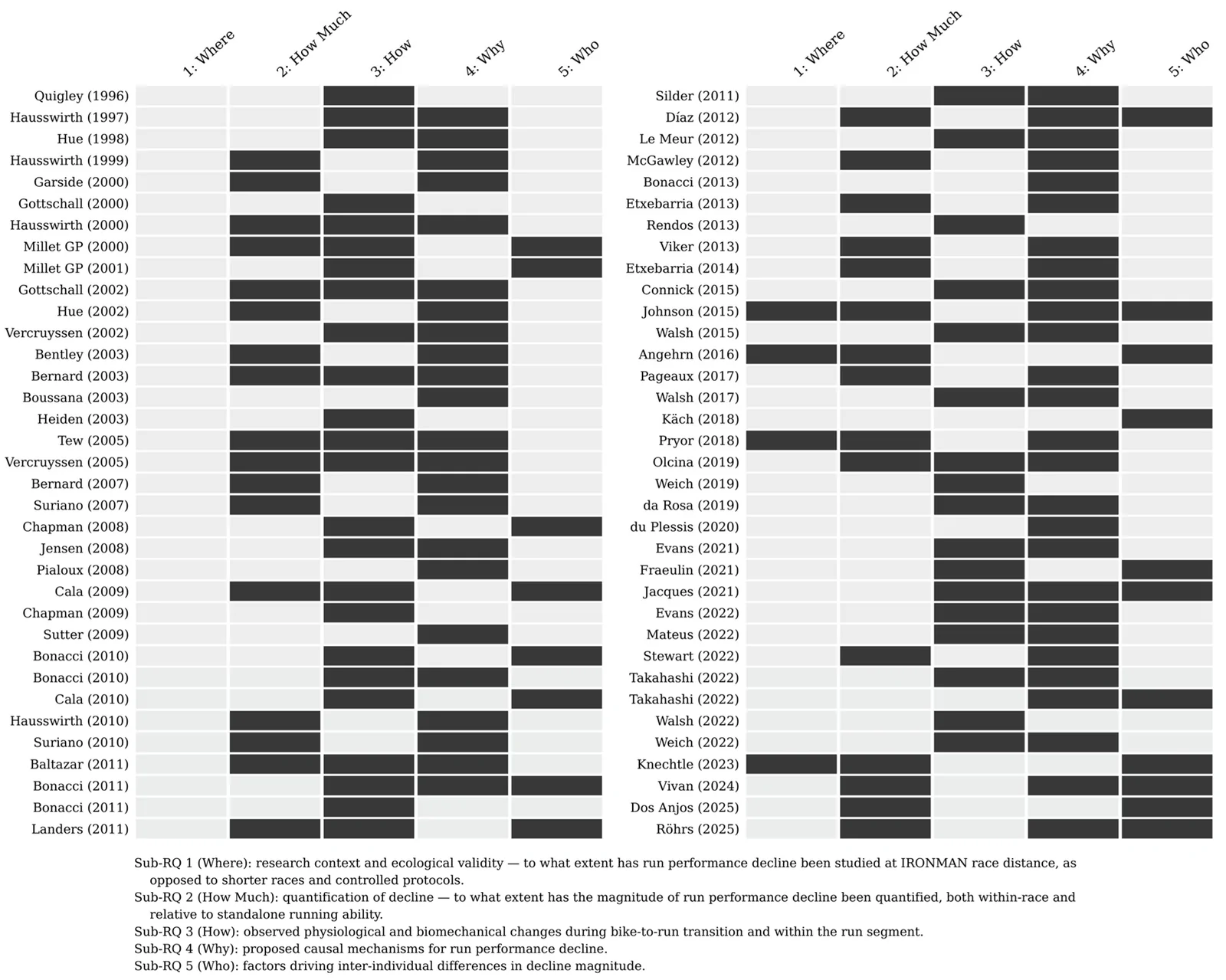
Sub-RQ mapping profile cross-tabulation of studies by sub-questions, showing all 70 included studies. Filled cells indicate that a study was tagged with the corresponding sub-question. Definitions of the sub-questions are provided in the figure footer. The studies shown correspond to references [10,11,12,13,14,15,16,17,18,19,20,21,22,23,24,25,26,27,28,29,30,31,32,33,34,35,36,37,38,39,40,41,42,43,44,45,46,47,48,49,50,51,52,53,54,55,56,57,58,59,60,61,62,63,64,65,66,67,68,69,70,71,72,73,74,75,76,77,78,79].

Sub-question tags were distributed as follows: Sub-RQ 1, 4 studies; Sub-RQ 2, 32; Sub-RQ 3, 41; Sub-RQ 4, 50; and Sub-RQ 5, 19 (Figure 2). Many studies have addressed more than one sub-question, with Sub-RQs 2 and 4 being the most frequently paired. Sub-RQ 4 had the highest overall count because the experimental literature is heavily focused on underlying physiological and biomechanical mechanisms. Sub-RQ 1 was assigned only to studies that analysed how athletes ran a full-distance IRONMAN race. Analyses of group averages, such as the top 100 finishers, were included as well as analyses of individual athletes. Studies that used IRONMAN race records only to compare run times between age groups were tagged with Sub-RQ 5 instead.

The studies included in this scoping review were published between 1996 and 2025. Of the 70 included studies, five analysed official IRONMAN race records, three took direct measurements of running during competition, and 62 used controlled bike-to-run protocols; of the latter, 21 were conducted in the field and 41 in the laboratory. Most controlled studies compared a run performed after cycling with a run performed without preceding cycling, using a randomised crossover or a repeated-measures design (Supplementary Table S1).

### 3.3. Sub-RQ 1: Research Context and Ecological Validity

Sub-RQ 1 asks to what extent running performance decline has been studied at the IRONMAN race distance, as opposed to shorter races and controlled protocols.

Four studies were tagged with Sub-RQ 1, all of which analysed how athletes ran a full-distance IRONMAN race. Knechtle et al. [10] and Angehrn et al. [11] used official race databases, covering the 2022 IRONMAN World Championship and 11 IRONMAN races held in 2014, respectively. Johnson et al. [12] and Pryor et al. [13] combined race timing data with measurements recorded on the athletes during the race, including heart rate, power, and positional data. None of the four compared IRONMAN running performance with the same athletes’ standalone running ability. Their findings are described in Section 3.4.

Five of the 70 included studies used IRONMAN full-distance race data. Four of these analysed how athletes ran the race and were tagged with Sub-RQ 1; the fifth (Käch et al. [14]) used IRONMAN race records only to compare run times between age groups, and is therefore discussed under Sub-RQ 5. The remaining 65 studies were conducted at shorter race distances or under controlled protocols. Three recorded running movement directly during Olympic-distance competition [15,16]; the other 62 used controlled bike-to-run protocols in which the cycling segment was far shorter than an IRONMAN bike leg. None of the included studies examined running performance decline as its primary research question.

### 3.4. Sub-RQ 2: Quantification of Decline

Sub-RQ 2 concerned the quantification of running performance decline: how much decline has actually been measured and reported.

#### 3.4.1. Within-Race Pacing Decline

Within-race decline in running speed has been reported in IRONMAN races. Angehrn et al. [11] analysed the top 100 finishers of 11 races and found that running speed decreased progressively from the start to the end of the marathon in every race, at a rate of 0.06 to 0.34 km/h per split. Knechtle et al. [10] analysed 25 marathon splits among the top 30 professional finishers at the 2022 IRONMAN World Championship and found the same pattern in all groups, with the decline steeper in slower finishers. A related but distinct measure was used by Johnson et al. [12], who quantified how far athletes fell short of their own pre-race goal pace across 11 run segments; the run showed the largest shortfall of the three disciplines, and higher-placed athletes deviated far less than lower-placed athletes. In a single-athlete case study, Pryor et al. [13] found no significant change in velocity or normalised graded pace between laps of an IRONMAN won on a course with major elevation changes.

#### 3.4.2. Experimental Decline Measures

In the experimental literature, several studies have measured the decline in running performance after cycling. Olcina et al. [17] found that the 12 min run distance dropped by 5.8% after a 20 min cycling time trial compared to an isolated run. Suriano et al. [18] reported that the run time to exhaustion at 85% of maximal aerobic velocity was 40% longer following variable-intensity cycling than following constant-intensity cycling at the same mean power. This suggests that cycling strategy may influence the magnitude of performance decline. Tew [19] reported that a 10 km run time was significantly impaired after 65 min of cycling, and Bernard et al. [20] reported a 3 km run decline following a cycling segment.

#### 3.4.3. The Between-Context Gap

Despite extensive research on pacing and experimentally induced performance decline, we identified no study that quantified the “between-context decline,” defined as the difference between an individual athlete’s standalone marathon ability and their actual IRONMAN run split. The data required to perform this analysis are largely available. Marathon PBs are publicly available, IRONMAN results databases are extensive, and scatter plots in prediction studies (e.g., [4]) already plot marathon PB against IRONMAN runtimes. These plots visually indicated that athletes with very similar marathon PBs could exhibit markedly different IRONMAN running performances, with substantial scatters observed around the regression line. However, we identified no study that quantified this performance decline at the individual level. Further, the studies identified by this review provide no evidence regarding the mean magnitude of between-context decline, its variability across individuals, its distribution, or the factors associated with inter-individual differences in decline. These fundamental descriptive aspects remain unaddressed.

### 3.5. Sub-RQ 3: Observed Changes During and After Transition

The third sub-question asked what physiological and biomechanical changes have been observed during the bike-to-run transition and within the run segment.

In the experimental literature, elevated perceived exertion during post-cycling running is the most consistent finding. Other variables, such as stride parameters, cardiorespiratory measures, and joint kinematics, have shown inconsistent patterns across studies, reflecting differences in cycling protocols, running test conditions, and participant performance levels [6].

Several specific findings from the included studies illustrated these patterns. For spatiotemporal parameters, da Rosa et al. [21] reported that middle-level triathletes exhibited shorter stride lengths and higher stride frequency when running after cycling than during isolated running (2.60 vs. 2.65 m and 1.46 vs. 1.43 Hz, respectively), while the mechanical efficiency ratio remained unchanged despite a ~12% increase in metabolic cost. Connick and Li [22] reported a 45% increase in stride time variability (Cohen’s d = 0.95) at the onset of transition running after 3 h of cycling, accompanied by shorter stride lengths and transient reductions in ankle and hip range of motion that recovered within 10 min. Gottschall and Palmer [23] demonstrated that running stride frequency closely tracked prior cycling cadence; high-cadence cycling (+20%) produced a 10% higher running stride frequency, which was attributed to neural firing pattern perseveration. Overall, a decrease in stride length is accompanied by an increase in stride during transition running.

For neuromuscular control parameters, Bonacci et al. [24] reported that elite international triathletes, including four Olympians and a World Champion, preserved both electromyography patterns and sagittal plane joint angles after cycling at both low and high intensities, with only one of seven athletes (14%) showing any alteration in muscle recruitment. In contrast, 46% of moderately trained athletes exhibited altered neuromuscular patterns [25].

For running economy and metabolic responses, da Rosa et al. [21] reported that cost-of-transport increased significantly after cycling (3.71 vs. 3.31 J·kg ^−1^·m ^−1^); however, the total mechanical work and mechanical efficiency were maintained through compensatory stride adjustments. Bonacci et al. [24] reported only minor changes in running economy among elite athletes.

Evidence obtained during competition is limited and not consistent. Landers et al. [15] filmed 51 elite men during an Olympic-distance World Championship and found that stride length was significantly shorter 1.2 km after the transition than during the middle of the 10 km run, and shorter again before the finish, indicating both a transition effect and a fatigue effect; stride length, but not stride rate, was associated with run and overall performance. Cala et al. [16] analysed the 10 km run of an elite Olympic-distance World Cup triathlon and found no change in running kinematics during the first lap after the cycling segment; stride length and velocity declined only in the final lap, which the authors attributed to fatigue accumulated during the run rather than to the preceding cycling. The same study noted that running velocities during competition were substantially faster than those used in laboratory bike-to-run protocols.

What is noteworthy across these studies is that their results were inconsistent. Whether stride length increases or decreases and whether running economy worsens or remains the same depends on the cycling protocol, running test conditions, and, particularly, the athlete’s performance level. There is no single typical physiological or biomechanical transition response, and the few observations made during actual competition do not necessarily reproduce the responses reported in the laboratory.

### 3.6. Sub-RQ 4: Identified Causes and Mechanisms

Sub-RQ 4 examined the identified mechanisms proposed to explain why running worsens after cycling.

In the experimental literature, neuromuscular fatigue is the most commonly proposed explanation for the decline in running performance after cycling. However, the specific causal pathway (peripheral muscle fatigue, central fatigue, glycogen depletion, or biomechanical compensation) remains unclear, as studies using different protocols have produced conflicting results.

#### 3.6.1. Cycling Intensity and Strategy

Among the charted studies, Suriano and Bishop [26] showed that higher cycling intensity was associated with greater run impairment. Suriano et al. [18] reported that variable-intensity cycling ending at a low power improved subsequent runtime to exhaustion by 40% compared with constant-intensity cycling at the same average power, despite similar average physiological responses. This suggests that the metabolic state in the final minutes before the transition may be more important than the average effort during the entire ride.

#### 3.6.2. Nutritional Factors and Glycogen Depletion

McGawley et al. [27] reported that carbohydrate intake during cycling improves subsequent running, providing direct evidence that nutrition affects subsequent running performance. Glycogen depletion has often been cited as a contributing factor to the progressive decline in running performance observed in ultra-endurance events, as athletes are unable to fully replace the energy expended during racing [1].

#### 3.6.3. Evidence from Non-IRONMAN Protocols

In a sprint-distance field study, Hausswirth et al. [28] showed that drafting during cycling, which reduced $\dot{V}$O_2_ by 14% and halved blood lactate, led to improved subsequent 5 km running performance. Although drafting is not allowed in IRONMAN races, this finding supports the hypothesis that lower energy expenditure during cycling may contribute to improved subsequent running performance.

It remains unclear whether these mechanisms operate similarly during an actual IRONMAN race, where athletes accumulate fatigue over 8–17 h under conditions of progressive glycogen depletion, heat stress, and nutritional challenge that have not been fully replicated in laboratory protocols. Consequently, the mechanisms underlying running performance decline under real IRONMAN conditions remain insufficiently investigated.

### 3.7. Sub-RQ 5: Factors Underlying Inter-Individual Variability

The fifth sub-question asked what factors drive inter-individual differences in the magnitude of running performance decline.

#### 3.7.1. Performance Level as a Moderator

A higher performance level appeared to reduce the likelihood of altered neuromuscular control during the bike-to-run transition [24,25,29]. Within a single study, Millet et al. [30] demonstrated that elite triathletes reduced their oxygen cost by 3.7% after cycling, whereas middle-level triathletes exhibited a 2.3% increase in oxygen cost. These results suggest that performance level moderates transition quality, although the underlying reason remains unclear.

#### 3.7.2. Age and Sex

Several included studies examined sex differences in the response to prior cycling, with mixed results. Vivan et al. [31] found that 5 km time-trial performance was significantly impaired after cycling at 90% of functional threshold power compared with 80% in men, but not in women, which the authors attributed to differences in substrate use and muscle fibre composition. Cala et al. [16] reported that stride length and velocity declined across the run in men but were maintained in women during a World Cup race. In contrast, Angehrn et al. [11] found no sex difference in the pacing pattern of the run across 11 IRONMAN races, and Knechtle et al. [10] found that men and women differed in the variability of their running pace rather than in its overall trajectory.

Evidence on age is more limited. Käch et al. [14] reported that age-related decline in running began in the 30–34 year age-group in women and the 35–39 year group in men, and that the sex difference in IRONMAN run times has narrowed among professional athletes but widened in the 40–49 year age groups. This describes decline with age rather than decline caused by prior exertion; we identified no study that examined whether age modifies the magnitude of prior exertion-induced running performance decline.

#### 3.7.3. Prediction of Overall Performance

Beyond the formal scope of this review, the broader IRONMAN literature has identified predictors of the overall finish time. Marathon PB is consistently among the strongest predictors of IRONMAN performance [4,5,80], along with Olympic-distance triathlon PB, weekly training volume, and body composition. Knechtle et al. [1] comprehensively summarised these predictors, and a more recent analysis of 687,696 age-group finishers [2] confirmed and extended these findings.

#### 3.7.4. Analytical Framing in Existing Prediction Studies

One gap identified by this review is that the prediction literature has focused primarily on predicting overall finish time or run split time, rather than quantifying how far running performance differs from an athlete’s standalone marathon performance. Marathon PB is one of the strongest predictors of IRONMAN performance, but we identified no study that used it as a baseline against which to measure performance decline. The simple calculation of “IRONMAN run split minus expected marathon time”—asking what predicts that difference—was not attempted in any study identified by this review. Scatter plots in prediction studies (e.g., [4]) already show substantial variability around the regression line, indicating that athletes with similar marathon PBs can exhibit markedly different IRONMAN running performance. The factors underlying this variability remain unclear but may include differences in training history, cycling intensity, nutritional strategy, age, body composition, and pacing. Identifying the determinants of this variability could have important implications for coaching and race preparations. However, this variability was not formally quantified in the studies identified by this review, and none of them examined its predictors.

## 4. Discussion

### 4.1. Summary of Principal Findings

This scoping review examined 70 studies related to running performance decline in IRONMAN-distance triathlon. The remaining records retrieved by the search were largely concerned with related but distinct topics, such as overall race time predictors, demographic comparisons, non-IRONMAN race data, and laboratory studies without measures of running performance. Despite substantial research interest in IRONMAN performance, the studies identified by this review did not directly investigate running performance decline as their main research question.

Regarding the source of the evidence (Sub-RQ 1), only four of the 70 included studies analysed how running performance changed over the course of a full-distance IRONMAN race, and even these characterised decline in different ways: from split-by-split running speed, from deviation from an athlete’s own goal pace, and from lap-to-lap variation in a single athlete. Of the remaining studies, one used IRONMAN race records to compare run times between age groups rather than to analyse the run itself, and the rest were conducted at shorter race distances or under controlled protocols in which the cycling segment was far shorter than an IRONMAN bike leg.

Regarding the extent of running performance declines (Sub-RQ 2), the review identified a major gap in the literature. Within-race pacing slowdowns have been described for groups of athletes, and controlled bike-to-run studies have reported approximately 2–6% decline in short transition runs. However, between-context decline, defined as the difference between an athlete’s standalone marathon performance and their IRONMAN running performance, was not quantified in any study identified by this review. Consequently, basic descriptive statistics regarding the magnitude, distribution, and variability of this measure are currently unavailable.

Regarding physiological and biomechanical changes (Sub-RQ 3), the evidence shows a wide range of responses during the bike-to-run transition, but the findings are inconsistent across studies. Stride adjustment and higher perceived exertion were the most reproducible findings.

For the reasons behind performance decline (Sub-RQ 4), neuromuscular fatigue was the most frequently proposed mechanism; however, the evidence is inconsistent, and the specific causal pathway remains unclear. Cycling intensity and nutrition have some empirical support as modifiable factors, although this evidence comes from short protocols that do not replicate IRONMAN conditions, and the few measurements made during actual competition do not consistently reproduce the responses reported in the laboratory.

For the factors driving inter-individual variability (Sub-RQ 5), the evidence suggests that elite athletes are less affected by cycling-induced fatigue than age-group athletes. Many variables predict the overall IRONMAN race time, but no study identified by this review investigated the magnitude of decline relative to standalone running ability. This shift in analytical framing—from predicting race time to quantifying performance decline—was not observed in the studies identified by this review.

### 4.2. Implications for Real-Race Research

Taken together, the identified gaps highlight several priorities for future research. In particular, studies using real IRONMAN race data are needed to: (i) define running performance decline relative to each athlete’s baseline running ability; (ii) quantify the magnitude and variability of this decline; and (iii) identify factors associated with inter-individual differences in decline.

Three practical difficulties help explain why the between-context decline was not quantified in any study identified by this review, and each has implications for study design. First, a standalone reference performance is required, and marathon PB is an imperfect proxy: it may have been recorded under different course and environmental conditions, at a different point in the training cycle, or several years earlier. Laboratory-derived surrogates such as lactate threshold pace or shorter time-trial results carry their own limitations, because prediction models based on population averages cannot capture individual endurance profiles. A reference performance should therefore be both recent and reasonably comparable in course and conditions, and future studies should specify and report the eligibility window they apply. Second, replicating a full IRONMAN race in the laboratory—3.8 km swimming, 180 km cycling, then 42.2 km running—is not feasible as an experimental protocol; existing protocols, typically 20–50 min of cycling, are far shorter than the demands of an actual race. Third, real-race data are subject to numerous uncontrolled variables, including temperature, course profile, nutrition, pacing decisions, training status, and the athlete’s goals for the day. Although these limitations are real, real-race data may nevertheless provide valuable opportunities for investigating research questions that are difficult to address under controlled experimental conditions.

Given this, age-group athletes warrant particular attention. They constitute the vast majority of IRONMAN finishers, with the largest participation observed among men aged 40–44 years [3,81]. Unlike professionals, age-group athletes bring diverse training backgrounds, racing experiences, and physiological capacities to the starting line. This diversity provides an opportunity to investigate inter-individual variations in running performance decline, as the broader range of training backgrounds, racing experience, and physiological characteristics may facilitate the identification of factors associated with differences in performance decline.

The practical implications of such studies are significant. If we can characterise running performance decline and identify its predictors, this knowledge could be used for pre-race benchmarking, setting realistic run split expectations based on individual ability, as well as for optimising pacing strategy, nutrition planning, and identifying athletes who are at high risk of severe decline. Such knowledge could facilitate the development of targeted training and race-day interventions.

### 4.3. Limitations of This Scoping Review

This review has some limitations that should be acknowledged. Title and abstract screening was performed by a single reviewer (Y.T.) with the assistance of the active-learning algorithm in ASReview LAB, and the eligibility criteria were agreed with H.N. before screening, but the individual screening decisions were not independently verified by a second reviewer; this may have introduced selection bias. The active-learning procedure ranks records by estimated relevance and, under the pre-specified stopping rule, screening ended once 50 consecutive records had been labelled as not relevant. As a result, 659 of the 896 records (73.5%) were ranked below the stopping point and were not individually screened, so relevant studies may remain among them. To partially mitigate this, the labelled records were checked after screening against a set of studies known to the review team; this check identified one eligible study that had not been presented during active-learning screening, which was then screened and included. This finding indicates that the active-learning approach was highly effective, although the possibility that a small number of eligible studies remained unscreened cannot be excluded.

Searches were limited to two databases (PubMed and SPORTDiscus). Other databases such as Scopus, Web of Science, and Embase were not searched and may index relevant studies not retrieved here. Although no language restriction was applied, English-only search terms may have missed studies published in other languages. Following the standard scoping review methodology, we did not conduct formal quality appraisals of the included studies, and the goal was to map the scope of the available evidence and not evaluate its quality. Among the 11 excluded studies, two were excluded owing to inaccessibility of the full text; we did not contact the authors of those papers, so relevant data may have been missed.

Limiting observational studies to IRONMAN full distance excluded data from middle-distance, Olympic, and other triathlon formats that might provide relevant evidence. However, this restriction was considered necessary because only the full IRONMAN distance includes a marathon-length run performed after several hours of prior exertion, which was the focus of the present review. We also excluded experimental studies measuring only physiological cost variables at fixed running speeds, rather than studies that quantitatively addressed running performance decline or movement pattern changes. As a consequence of this narrower review scope, some mechanism-focused evidence from shorter-distance experimental studies was not considered in detail in this review.

### 4.4. Recommendations for Future Research

Based on the gaps identified in this scoping review, the following research priorities are proposed:

(1) Quantify between-context decline. Future studies should calculate and report the differences between standalone marathon ability (or a validated proxy) and IRONMAN running performance at the individual level. The difference can be expressed in more than one way, and each choice has consequences: an absolute time difference preserves the units in which race outcomes are decided; a relative difference or ratio allows athletes of different ability to be compared on a common scale; and a log-transformed difference yields a more symmetric distribution that is better suited to regression modelling. Studies should state which form they use and why and should also state how recent and how comparable the reference performance must be. Reporting descriptive statistics of this difference (mean, standard deviation, range, and distribution shape) would help characterise its magnitude and variability, aspects that were not described in the studies identified by this review.

(2) Treat inter-individual variability as a primary outcome. Rather than reporting group means alone, future studies should explicitly model and explain individual differences in the magnitude of decline. Multivariate regression incorporating marathon PB, training volume, cycling intensity, pacing strategy, age, sex, and environmental conditions would help estimate the contribution of each factor. Although existing studies have focused primarily on predicting total race time, we identified no study that quantified or modelled performance decline itself.

(3) Conduct observational studies using real IRONMAN race data. Large-scale analyses linking race results with athletes’ marathon PBs and race-day variables would directly address the gaps identified above. Although these studies face methodological challenges, such as defining a valid running baseline and controlling for confounding variables (e.g., weather, course profile, and race nutrition), these challenges may be addressed through appropriate statistical methods.

(4) Focus on age-group athletes. Given that they comprise the majority of the IRONMAN participants and show the greatest performance variability, this population requires dedicated research attention.

(5) Bridge the laboratory-to-field gap. Mechanism studies may benefit from using protocols that more closely approximate IRONMAN durations and conditions or from evaluating whether findings obtained from shorter protocols generalise to real-race settings.

## 5. Conclusions

This scoping review of 70 studies shows that the literature on running performance decline in IRONMAN-distance triathlon has made progress in several areas: pacing patterns have been described, performance predictors have been identified, and laboratory studies have begun to clarify the physiological effects of the bike-to-run transition. However, these research streams have not addressed key questions relating to individual variation in running performance decline. In particular, the difference between IRONMAN running performance and standalone marathon performance, and the factors associated with inter-individual variation in that difference, were not systematically investigated in the studies identified by this review. Basic descriptive statistics of between-context decline, such as mean, variance, and distribution, were not reported. Individual differences that are clearly visible in existing scatter plots were not analytically pursued. Mechanistic evidence comes almost entirely from short controlled protocols that cannot replicate the conditions of an actual IRONMAN race, and the few measurements made during competition do not consistently reproduce laboratory findings.

Thus, observational studies using real IRONMAN race data are needed to quantify between-context decline at the individual level and identify factors associated with inter-individual variation in decline. Age-group athletes may represent a particularly relevant population for such investigations, as they constitute the majority of IRONMAN participants and exhibit substantial performance heterogeneity.

## Data Availability

No new data were created or analysed in this study. The included studies and charted data are available from the corresponding author upon reasonable request. The search strings used to identify the included studies are listed in Supplementary Table S2.

## Supplementary Materials

The following supporting information can be downloaded at https://www.mdpi.com/article/10.3390/sports14080345/s1. Table S1: Characteristics of all the included studies (*n* = 70); Table S2: Complete search strings by database; Table S3: Studies excluded at full-text screening (*n* = 11) with reasons for exclusion. File: Preferred Reporting Items for Systematic reviews and Meta-Analyses extension for Scoping Reviews (PRISMA-ScR) Checklist. References [10,11,12,13,14,15,16,17,18,19,20,21,22,23,24,25,26,27,28,29,30,31,32,33,34,35,36,37,38,39,40,41,42,43,44,45,46,47,48,49,50,51,52,53,54,55,56,57,58,59,60,61,62,63,64,65,66,67,68,69,70,71,72,73,74,75,76,77,78,79] are cited in the Supplementary Materials.
