# Supplementary material for: Running Performance Decline in IRONMAN-Distance Triathlon: A Scoping Review of Existing Evidence and Research Gaps
Source: Sports (Basel). 2026 Aug 10;14(8):345. doi: 10.3390/sports14080345 (PMC13517492; doi:10.3390/sports14080345)
Supplement: Supplementary file 1 [file sports-14-00345-s001.zip › sports-4449193-checklist.pdf]

Preferred Reporting Items for Systematic reviews and Meta-Analyses extension for Scoping Reviews (PRISMA-ScR) Checklist

Manuscript: *Running Performance Decline in IRONMAN-Distance Triathlon: A Scoping Review of Existing Evidence and Research Gaps*

Authors: Yujiro Tsutsumi, Shuichi Machida, and Hisashi Naito

SECTION	ITEM	PRISMA-ScR CHECKLIST ITEM	REPORTED IN SECTION / NOTE
**TITLE**			
Title	1	Identify the report as a scoping review.	Title page (Title contains "A Scoping Review")
**ABSTRACT**			
Structured summary	2	Provide a structured summary that includes (as applicable): background, objectives, eligibility criteria, sources of evidence, charting methods, results, and conclusions that relate to the review questions and objectives.	Abstract
**INTRODUCTION**			
Rationale	3	Describe the rationale for the review in the context of what is already known. Explain why the review questions/objectives lend themselves to a scoping review approach.	Section 1 (Introduction)
Objectives	4	Provide an explicit statement of the questions and objectives being addressed with reference to their key elements (e.g., population or participants, concepts, and context) or other relevant key elements used to conceptualize the review questions and/or objectives.	Section 1 (Introduction; primary research question and five sub-questions)
**METHODS**			
Protocol and registration	5	Indicate whether a review protocol exists; state if and where it can be accessed (e.g., a Web address); and if available, provide registration information, including the registration number.	Section 2.1 (No formal protocol was pre-registered; rationale stated in Methods)
Eligibility criteria	6	Specify characteristics of the sources of evidence used as eligibility criteria (e.g., years considered, language, and publication status), and provide a rationale.	Section 2.2 (Eligibility criteria)
Information sources	7	Describe all information sources in the search (e.g., databases with dates of coverage and contact with authors to identify additional sources), as well as the date the most recent search was executed.	Section 2.3 (Search strategy)
Search	8	Present the full electronic search strategy for at least 1 database, including any limits used, such that it could be repeated.	Section 2.3 and Supplementary Table S2 (Complete search strings)
Selection of sources of evidence	9	State the process for selecting sources of evidence (i.e., screening and eligibility) included in the scoping review.	Section 2.4 (Source selection)
Data charting process	10	Describe the methods of charting data from the included sources of evidence (e.g., calibrated forms or forms that have been tested by the team before their use, and whether data charting was done independently or in duplicate) and any processes for obtaining and confirming data from investigators.	Section 2.5 (Data charting)

Data items	11	List and define all variables for which data were sought and any assumptions and simplifications made.	Section 2.5 and Supplementary Table S1 (charting fields)
Critical appraisal of individual sources of evidence (optional)	12	If done, provide a rationale for conducting a critical appraisal of included sources of evidence; describe the methods used and how this information was used in any data synthesis (if appropriate).	Not applicable (critical appraisal is not required for scoping reviews and was not performed)
Synthesis of results	13	Describe the methods of handling and summarizing the data that were charted.	Section 2.5 (synthesis approach using five sub-questions)
**RESULTS**			
Selection of sources of evidence	14	Give numbers of sources of evidence screened, assessed for eligibility, and included in the review, with reasons for exclusions at each stage, ideally using a flow diagram.	Section 3.1 and Figure 1 (PRISMA-ScR flow diagram)
Characteristics of sources of evidence	15	For each source of evidence, present characteristics for which data were charted and provide the citations.	Section 3.2 and Supplementary Table S1 (Characteristics of all included studies)
Critical appraisal within sources of evidence (optional)	16	If done, present data on critical appraisal of included sources of evidence (see item 12).	Not applicable (no critical appraisal performed)
Results of individual sources of evidence	17	For each included source of evidence, present the relevant data that were charted that relate to the review questions and objectives.	Sections 3.3–3.7 (Sub-RQ 1–5) and Supplementary Table S1; Figure 2 (Sub-RQ mapping)
Synthesis of results	18	Summarize and/or present the charting results as they relate to the review questions and objectives.	Sections 3.3–3.7 (Sub-RQ 1–5)
**DISCUSSION**			
Summary of evidence	19	Summarize the main results (including an overview of concepts, themes, and types of evidence available), link to the review questions and objectives, and consider the relevance to key groups.	Section 4.1 (Summary of principal findings) and Section 4.2 (Implications for real-race research)
Limitations	20	Discuss the limitations of the scoping review process.	Section 4.3 (Limitations of this scoping review)
Conclusions	21	Provide a general interpretation of the results with respect to the review questions and objectives, as well as potential implications and/or next steps.	Section 5 (Conclusions) and Section 4.4 (Recommendations for future research)
**FUNDING**			
Funding	22	Describe sources of funding for the included sources of evidence, as well as sources of funding for the scoping review. Describe the role of the funders of the scoping review.	Funding section (back matter): " This study was supported by Faculty of Health and Sports Science and the Institute of Health and Sports Science & Medicine, Juntendo University."

JBI = Joanna Briggs Institute; PRISMA-ScR = Preferred Reporting Items for Systematic reviews and Meta-Analyses extension for Scoping Reviews.

From: Tricco AC, Lillie E, Zarin W, O'Brien KK, Colquhoun H, Levac D, et al. PRISMA Extension for Scoping Reviews (PRISMA-ScR): Checklist and Explanation. ***Ann Intern Med.*** *2018;169(7):467–473. doi:10.7326/M18-0850.*
