# Supplementary material for: Running Performance Decline in IRONMAN-Distance Triathlon: A Scoping Review of Existing Evidence and Research Gaps
Source: Sports (Basel). 2026 Aug 10;14(8):345. doi: 10.3390/sports14080345 (PMC13517492; doi:10.3390/sports14080345)
Supplement: Supplementary file 1 [file sports-14-00345-s001.zip › Supplementary_with_ref_column.pdf]

Supplementary Materials for:

Running Performance Decline in IRONMAN-Distance Triathlon: A Scoping Review of Existing Evidence and Research Gaps

Yujiro Tsutsumi, Shuichi Machida, and Hisashi Naito

Supplementary Table S1. Characteristics of all included studies (n = 70).

Author	Year	Ref.	Study Type	Design	Sample	Level	Age	Sub-RQs	Main Finding
Quigley and Richards	1996	[34]	Controlled bike-to-run (laboratory)	Repeated measures (3 conditions)	11 (11M)	Recreational triathletes/ duathletes	Not reported	3	Three-dimensional kinematic and kinetic analysis showed no significant differences in stance time, flight time, step length, vertical displacement of the center of gravity, or sagittal hip/knee/ankle kinematics and kinetics between control and post-cycling runs. The authors concluded that bike-to-run discomfort may be attributable to motor control or physiological factors rather than detectable mechanical changes at this cycling intensity.
Hausswirth et al.	1997	[35]	Controlled bike-to-run (laboratory)	Repeated measures (3 trials)	7 (7M)	Well-trained triathletes	Not reported	3, 4	Energy cost during the marathon run was significantly higher than during the triathlon run (+3.2%, $p < 0.05$) and the isolated run (+11.7%, $p < 0.01$), with both the triathlon and marathon associated with greater body mass loss (1.6 ± 0.02 kg, $p < 0.01$) than the isolated run (0.7 ± 0.2 kg). After cycling, triathletes adopted a more forward-leaning posture, while the trunk gradient was less marked during the marathon; knee extension at foot-strike and maximal knee angle in the non-support phase both increased during the marathon run compared to the triathlon and isolated runs. The authors concluded that no single kinematic variable could fully explain the decrement in running efficiency, suggesting that running economy at the end of a triathlon and a marathon reflects global alterations of multiple parameters rather than any single mechanism.
Hue et al.	1998	[36]	Controlled bike-to-run (laboratory)	Repeated measures (4 phases)	7 (7M)	Competitive triathletes (VO_2max 62.1 mL·kg^{-1}·min^{-1})	Not reported	3, 4	Cycle-to-run transition elicited elevated VO_2, VE, HR, VE/VO_2, and breathing frequency during the first 7 min of the run segment compared to an isolated control run. The findings established that the bike-to-run transition imposes transient metabolic and cardiorespiratory disturbance during the initial post-cycling phase.
Hausswirth et al.	1999	[28]	Controlled bike-to-run (field)	Repeated measures (2 conditions)	8 (8M)	Elite international (French & Finnish national team)	20.8±2.1	2, 4	Drafting during the cycling leg of a sprint triathlon reduced energy expenditure by ~14% VO2 and halved blood lactate, enabling significantly faster subsequent 5-km run performance (+5.5% TA vs IR narrowed to +1.8% TD vs IR). Strongest runners benefited most from drafting.
Garside and Doran	2000	[37]	Controlled bike-to-run (laboratory)	Counterbalanced crossover	8 (8M)	Olympic/IRONMAN-experienced triathletes (3–11 yrs)	25.3±5.1	2, 4	A steeper seat tube angle (81°) compared to the standard shallow angle (73°) during a 40-km cycle at ~70% VO_2peak significantly improved subsequent 10-km running time (42:55 ± 4:19 vs. 46:15 ± 4:52 min, $p < 0.01$) and combined cycle-plus-run performance (1:45:49 ± 5:45 vs. 1:50:33 ± 6:08, $p < 0.001$), with the advantage almost entirely confined to the first 5 km of the run (21:41 ± 2:15 vs. 24:15 ± 2:31 min) and not evident in the second 5 km. Heart rate, stride length, and stride frequency were all increased during the 81° run ($p < 0.05$) despite equivalent submaximal VO_2, HR, and blood lactate responses during the cycle. The authors proposed that the steeper frame geometry attenuates the 'residual effect' of prior cycling by orienting the lower limb during cycling closer to its running position,

Author	Year	Ref.	Study Type	Design	Sample	Level	Age	Sub-RQs	Main Finding
									minimizing the alterations in muscular recruitment normally required at the bike-to-run transition.
Gottschall and Palmer	2000	[38]	Controlled bike-to-run (field)	Repeated measures (2 conditions)	10 (10M)	University-team triathletes	23±1	3	Step length after cycling was initially shorter and step frequency higher than after running at matched heart rate during the first 2 km of a 5-km race-effort run, with both parameters progressively converging across the remaining laps (condition × lap interactions, $p < 0.01$ for SL, $p < 0.0001$ for SF). Running efficiency (speed/HR) became significantly higher in the cycle/run condition by the conclusion of the 5-km run, despite similar mean speed across conditions. The authors interpreted the acute kinematic disturbances as perseveration of cycling cadence followed by gradual central pattern generator/motor program adjustment to the optimal running frequency.
Hausswirth et al.	2000	[39]	Controlled bike-to-run (laboratory)	Randomised crossover	7 M	Well-trained (Olympic-distance triathletes)	31±5	2, 3, 4	Prolonged run (2h15) induced greater neuromuscular alterations (elevated QiEMG, RMS +22%, MPF decrease) than triathlon of equal duration. Both TR and PR showed increased Ec vs IR, but triathlon induced less muscle fatigue than prolonged running alone, suggesting multi-discipline exercise distributes load across muscle groups.
Millet et al.	2000	[30]	Controlled bike-to-run (laboratory)	Pre-post, between-group (2 performance levels)	8 elite (1M/7F) + 18 middle-level (14M/4F)	Elite vs. middle-level triathletes (mixed comparison)	Not reported	2, 3, 5	Elite triathletes showed a trend toward reduced mechanical cost during the 7-min post-cycling run, while middle-level triathletes exhibited increased VO_2, VE, and HR. The performance-level-dependent physiological response suggests that elite triathletes better preserve metabolic efficiency through the bike-to-run transition than middle-level athletes.
Millet et al.	2001	[40]	Controlled bike-to-run (laboratory)	Pre-post, between-group (2 performance levels)	8 elite (1M/7F) + 18 middle-level (14M/4F)	Elite vs. middle-level triathletes (mixed comparison)	Not reported	3, 5	Middle-level triathletes showed increases in mechanical, kinetic, and potential energy cost during the first minute of post-cycling running, while elite triathletes showed smaller increases. The findings suggest that elite triathletes regulate spring-mass stiffness more effectively than middle-level triathletes during the bike-to-run transition onset.
Gottschall and Palmer	2002	[23]	Controlled bike-to-run (field)	Crossover, partially randomised (3 conditions)	13 (13M)	Trained (university triathlon team, 2+ yrs experience)	24.78±1.20	2, 3, 4	Fast cycling cadence (+20%) before running increased 3200-m run speed by 7% vs slow cadence, primarily through increased stride frequency with unchanged stride length and joint angles. Perseveration and central pattern generator effects are the proposed neural mechanisms for cadence-to-SF transfer.
Hue et al.	2002	[41]	Controlled bike-to-run (field)	Randomised controlled, 6 weeks (intervention)	12M (multibloc k n=6, normal n=6)	Competitive	M group: 29.0±3.1 yrs, 177.0±2.7cm, 71.0±2.3kg; N group: 29.7±3.5 yrs, 178.7±2.4cm, 72.2±2.6kg	2, 4	6 weeks of multicycle-run training did not improve overall cycle-run performance more than normal training in competitive triathletes. However, multiblock-trained group showed significantly better time during the first 333m run lap (bike-to-run transition onset), suggesting improved ability to handle the bike-to-run changeover specifically.

Author	Year	Ref.	Study Type	Design	Sample	Level	Age	Sub-RQs	Main Finding
Vercruyssen et al.	2002	[42]	Controlled bike-to-run (laboratory)	Randomised crossover	8 (8M / 0F)	Well-trained inter-regional triathletes	24.0±3.0 yrs	3, 4	Post-cycling running showed increased energy cost vs isolated run. VO2 slow component during running appeared only after higher cycling cadences (FCC, MOC), not after energetically optimal cadence (~73 rpm). EOC may be strategically beneficial.
Bentley et al.	2003	[43]	Controlled bike-to-run (laboratory)	Randomised crossover	8 (6M/2F)	Moderately trained triathletes (sprint to middle-distance triathlon)	26.2±3.4	2, 4	With adequate 5h recovery and nutrition, an incremental running test performed after maximal cycling produces equivalent results (vmax, VO2max, LT) to a control test without prior cycling. Maximum workload from cycling and running incremental tests are highly correlated (r=0.94-0.97) with 20km cycling and 5km running TT performance.
Bernard et al.	2003	[20]	Controlled bike-to-run (field)	Randomised crossover	9 (9M / 0F)	National-level triathletes	24.9±4.0 yrs	2, 3, 4	3km run impaired after 20-min cycling vs isolated run; no cadence effect on run time, but initial running velocity and stride rate transiently influenced by prior cadence. Subjects sustained higher VO2max fraction after low cadence.
Boussana et al.	2003	[44]	Controlled bike-to-run (laboratory)	Randomised crossover	10 (10M)	Competitive triathletes (≈5.6 yrs experience)	22.6±1.1	4	Moderate-intensity cycling at 75% VO_2max (20 min), but not running of equivalent duration and intensity, induced significant decreases in maximal inspiratory pressure (PImax: 132.4±4.9 → 125.7±5.6 cmH_2O post-C, $p < 0.05$) and respiratory muscle endurance time-limit (Tlim: 5.22±0.28 → 3.68±0.32 min post-C, $p < 0.05$). The cycling-induced respiratory muscle fatigue was maintained—neither reversed nor worsened—through a successive 20-min run (PImax: 133.7±4.0 → 126.9±5.2 cmH_2O; Tlim: 5.29±0.18 → 3.49±0.41 min, both $p < 0.05$), indicating that the crouched cycling position imposes a specific respiratory muscle load not present in upright running.
Heiden and Burnett	2003	[45]	Controlled bike-to-run (field)	Randomised crossover	10 triathletes	Trained triathletes (Olympic time ≈2h05min)	Not reported	3	Post-cycling running elicited increased vastus lateralis (VL) activation amplitude during stance phase, increased VL activation time during flight phase, and decreased biceps femoris (BF) activation amplitude during stance phase compared to control runs. The shift in quadriceps-hamstring coordination raises concerns regarding altered knee joint stability during the transition from non-weight-bearing cycling to weight-bearing running.
Tew	2005	[19]	Controlled bike-to-run (laboratory)	Randomised crossover	8 (8M / 0F)	Well-trained recreational triathletes	38.9±15.4 yrs	2, 3, 4	10km run significantly impaired after 65-min cycling vs isolated run; no cadence effect (72-97 rpm) on run performance despite transient effects on initial running velocity and biomechanical changes.
Vercruyssen et al.	2005	[46]	Controlled bike-to-run (laboratory)	Crossover	8 M	Well-trained (sprint/Olympic, ≥5 yr racing)	28.9±7.4	2, 3, 4	Low cadence (FCC-20%, ~74 rpm) during final 10 min of cycling significantly improved subsequent running time to fatigue by ~37-43% compared to freely chosen or high cadence. Benefit appears related to reduced muscular load during cycling rather than metabolic changes during running.
Bernard et al.	2007	[47]	Controlled bike-to-run (field)	Randomised crossover	10 (10M)	Trained triathletes (≥4 yrs experience)	25.2±6.8	2, 4	5-km running time was significantly faster after CI cycling (1118 ± 72 s) than after FCI (1134 ± 64 s, −1.4%) or VI (1168 ± 73 s, −4.4%) cycling (both $p < 0.05$), with FCI also faster than VI (−2.9%, $p < 0.05$); first-kilometre running velocity was

Author	Year	Ref.	Study Type	Design	Sample	Level	Age	Sub-RQs	Main Finding
									significantly higher after CI and FCI (4.57 m·s⁻¹) than after VI (4.35 m·s⁻¹, $p < 0.05$). Despite the substantial running-performance differences, no significant differences in VO_2, VE, HR, [La⁻], or RPE were observed either between the three cycling conditions or between the three subsequent running bouts, indicating that the metabolic load during cycling was matched across protocols. The authors concluded that neuromuscular factors—such as altered peripheral muscle recruitment or accumulated muscle fatigue from repeated power-output surges during the VI ride—rather than aerobic/anaerobic metabolic differences, likely explain the impaired running performance after variable-intensity cycling.
Suriano et al.	2007	[18]	Controlled bike-to-run (laboratory)	Randomised crossover	8 (8M)	Well-trained triathletes	28.9±7.4	2, 4	Variable-intensity cycling (ending at low power) improved subsequent run time to exhaustion by ~40% compared to constant cycling at same mean power output, despite similar average physiological responses during cycling. The reduced power output in the final 5 min likely allowed pre-transition recovery. Running economy was unaffected by cycling strategy.
Chapman et al.	2008	[29]	Controlled bike-to-run (laboratory)	Repeated measures (2 experiments)	14 triathletes	Highly-trained triathletes (≈6.6 yrs experience)	Not reported	3, 5	Tibialis anterior (TA) activation amplitude during stance phase was reduced after cycling in 5 of 14 triathletes, despite no overall group-level difference, indicating substantial individual variability in neuromuscular adaptation to the bike-to-run transition. The authors proposed that the altered TA activity reflects an effect of cycling on running motor commands rather than peripheral muscle fatigue.
Jensen et al.	2008	[48]	Controlled bike-to-run (laboratory)	Repeated measures (2 sub-studies)	Study 1: 11; Study 2: 9 (5 for EMG); sex not specified	Recreational/sub-elite triathletes	Study 1: 33.5±11.0; Study 2: 27.7±6.0	3, 4	Alternating seated/standing cycling in final 3 min produced lower HR and RPE during subsequent running vs all-seated cycling, but did not affect stride kinematics or EMG patterns. Stride changes (↑SF, ↓SL) after cycling were transient, normalizing within 2 min regardless of cycling posture.
Pialoux et al.	2008	[49]	Controlled bike-to-run (field)	Single-group pre-post	22 (22M)	Trained triathletes (≈5.5 yrs experience)	Not reported	4	Stride length and running speed showed no significant differences between control and post-cycling running. The authors concluded that under these moderate-intensity cycling conditions, post-cycling running biomechanics did not differ from isolated running.
Cala et al.	2009	[16]	Field measurement (during competition)	Cross-sectional	20 (10M/10F)	Elite triathletes — top 10 finishers from cycling portion at Triathlon World Cup	Not reported	2, 3, 5	First field-based kinematic analysis during World Cup triathlon. Prior cycling did not affect running kinematics at race start (Lap 1); performance decline (stride length, velocity) appeared only in final lap due to accumulated fatigue, not transition effect. Large velocity differences between lab and competition settings question external validity of lab-based transition studies. Men showed significant decline; women maintained technique throughout.
Chapman et al.	2009	[50]	Controlled bike-to-run (laboratory)	Repeated measures (repeatability, 2 sessions)	10 (6M/4F)	Highly-trained triathletes (≈6.8 yrs experience)	Not reported	3	This protocol paper established that the experimental procedure for measuring the direct effect of cycling on neuromuscular control during subsequent running is repeatable, with no differences in EMG or kinematics between control and

Author	Year	Ref.	Study Type	Design	Sample	Level	Age	Sub-RQs	Main Finding
									transition runs under the standardized protocol. The paper provides methodological foundation for subsequent transition studies.
Sutter et al.	2009	[51]	Controlled bike-to-run (laboratory)	Randomised crossover (3 conditions)	9 (6M/3F)	Well-trained triathletes	Not reported	4	Prior cycling intensity (below vs above BLa threshold) did not affect running economy (VO2) or vertical COM oscillation during subsequent treadmill running. However, VE and blood lactate were significantly affected by cycling intensity, with a significant VE-lactate correlation.
Bonacci et al.	2010	[52]	Controlled bike-to-run (laboratory)	Repeated measures	17 triathletes	Moderately-trained triathletes (≈4 yrs experience)	Not reported	3, 5	Moderately trained triathletes showed increased ankle dorsiflexion, more extended knee at ground contact, increased total knee ROM, and changes in muscle activity amplitude in 8 of 17 participants during post-cycling running, with ankle position at foot contact explaining 67.1% of variation in running economy. The findings indicate that neuromuscular control disturbances after cycling are individual-specific and biomechanically linked to running economy.
Bonacci et al.	2010	[25]	Controlled bike-to-run (laboratory)	Repeated measures (2 sessions)	15 triathletes	Moderately-trained triathletes (≈3 yrs experience)	Not reported	3, 4	Four of 15 moderately trained triathletes exhibited increased ankle dorsiflexion during ground contact in the first 5 min of transition running compared to control runs, while the remaining 11 participants showed preserved kinematics. The findings suggest moderately trained triathletes have variable difficulty in reproducing control-run kinematic patterns after cycling, with most adapting successfully and a subgroup not.
Cala et al.	2010	[53]	Field measurement (during competition)	Cross-sectional	16 F	Elite (World Cup)	Not reported	3, 5	In women's World Cup triathlon, the gold medalist exhibited significantly longer stride length, lower stride frequency, greater hip-toe cap distance, and more extended knee at toe-off compared to other top-16 finishers, potentially explaining performance differences in 10 km run.
Hausswirth et al.	2010	[54]	Controlled bike-to-run (field)	Randomised crossover (4 conditions)	10 M	Highly trained national-level triathletes (OD performance ~2h02±7min, ≥4 yrs training/competing)	24±3	2, 4	Best initial pacing strategy during OD triathlon run is 5% slower than 10km control run speed. A 20s variation in first 1km pacing led to 150±21s difference in overall 10km performance. Fast start (+5%) induced high metabolic demand and central down-regulation of pace. Too-slow start (-10%) prevented optimal pace adjustment due to deviation from prior experience template. First study to experimentally demonstrate pacing manipulation effects on triathlon run performance.
Suriano et al.	2010	[26]	Controlled bike-to-run (laboratory)	Repeated measures (multiple cycle intensities)	8 (5M/3F)	Trained triathletes (>2 yrs experience)	Not reported	2, 4	Running speed was significantly reduced during only the first 1 km of post-cycle running after 96-100% cycling intensity compared to control runs, with no differences in subsequent 4 km. No significant differences emerged in physiological markers (VO_2, HR, [La]) between control and transition runs, indicating that performance impairment was localized to the immediate post-transition phase and largely independent of overall physiological cost.

Author	Year	Ref.	Study Type	Design	Sample	Level	Age	Sub-RQs	Main Finding
Baltazar et al.	2011	[55]	Controlled bike-to-run (field)	Crossover	6 M	Amateur/recreational (3+ years competition)	24.1±7.8	2, 3, 4	Changing STA from 73° to 80° did not significantly alter cycling physiology but showed a trend toward improved subsequent 3 km run performance (P=0.065) and significantly increased stride frequency, suggesting steeper STA may reduce negative bike-to-run transition effects.
Bonacci et al.	2011	[24]	Controlled bike-to-run (laboratory)	Randomised crossover	7 (4M/3F)	Elite international (4 Olympians, 1 World Champion)	24.9±3.7	3, 4, 5	Elite international triathletes (including Olympians) preserved neuromuscular control and running economy after both low- and high-intensity cycling. Only 1/7 showed transient EMG change. Results confirm that elite athletes are substantially more resistant to cycling-induced running disruption than lower-level counterparts.
Bonacci et al.	2011	[56]	Controlled bike-to-run (laboratory)	Randomised controlled, 8 weeks (intervention)	15 screened; 8 randomised (those with aberrant NMC)	Moderately-trained triathletes	Not reported	3	Of 15 moderately-trained triathletes screened, eight exhibited aberrant neuromotor control during running after cycling and were randomised to 8 weeks of endurance-only versus endurance-plus-plyometric training. The combined plyometric and endurance training produced favourable neuromotor adaptations during running after cycling beyond those achieved by endurance training alone (number needed to treat = 2), with significant between-group differences at 8 weeks in the coefficient of multiple correlation ($p = 0.03$) and root mean square error ($p = 0.01$) of muscle activity patterns between control and transition runs. Running economy did not differ between groups at follow-up, indicating that plyometric training can correct altered neuromotor control during running after cycling without producing concurrent improvements in metabolic running economy.
Landers et al.	2011	[15]	Field measurement (during competition)	Cross-sectional	51 M	Elite (World Championship finishers)	Not reported	2, 3, 5	In elite male WC triathlon, stride length during the run was significantly correlated with run and overall performance, while stride rate was not. SL was reduced at the start and end of the run, suggesting transition and fatigue effects. Pre-T2 cadence increase ('spinning') did not correlate with initial run SR.
Silder et al.	2011	[57]	Controlled bike-to-run (laboratory)	Repeated measures (factorial)	14 (9M/5F)	Competitive triathletes (OD level: 40km bike ~1h12, 10km run ~43 min)	32±9	3, 4	Running demands significantly longer musculotendon lengths and more eccentric contractions than cycling across all lower extremity muscles. STA changes alone only reoriented body position (increased pelvic tilt) without altering joint kinematics, but increased RF activity. Hand position changes (aero→hoods) significantly increased hip extension, shifted uniarticular hip flexor/extensor muscle lengths closer to running ranges, and increased hamstring EMG. These combined effects may explain improved post-cycling running performance with triathlon-specific bike geometry.
Díaz et al.	2012	[58]	Controlled bike-to-run (field)	Longitudinal	15M (9 TID + 6 SE)	National TID (U16) & Senior Elite (Spanish National Squad)	TID: 15.2±0.7 → 16.2±0.7; SE: 24±5.6 → 24.8±5.6	2, 4, 5	Prior cycling impaired 3-km run performance in both TID and SE across 2 seasons, but TID improved longitudinally through pacing adaptation rather than cardiorespiratory changes. Field-based C-R test may detect specific T2 adaptations in developing athletes.

Author	Year	Ref.	Study Type	Design	Sample	Level	Age	Sub-RQs	Main Finding
Le Meur et al.	2012	[59]	Controlled bike-to-run (laboratory)	Repeated measures (3 sessions)	10 triathletes	Trained triathletes	Not reported	3, 4	Immediately after the 30-min cycling segment at ~80% VO_2max, leg stiffness was 12.1% higher than the control-run value with a concomitant increase in knee extensor EMG activity during pre-contact. Throughout the subsequent run to exhaustion at lactate threshold, leg stiffness decreased by 7.3%, while knee extensor and ankle flexor activities decreased during pre-contact and braking phases; no significant differences in spring-mass parameters or muscle activity were reported between the control run and the end of the transition run. The authors concluded that spring-mass behaviour changes during the cycle-run test were consistent with EMG activity changes, and that exercise cessation was not associated with significant residual alterations of stiffness values or EMG activity.
McGawley et al.	2012	[27]	Controlled bike-to-run (field)	Randomised single-blind crossover	10 (6M/4F)	Amateur	25±7 yrs; 66.8±9.2 kg (M: 26±8, 71.5±8.6kg; F: 24±5, 59.8±4.6kg)	2, 4	High-dose 2:1 maltodextrin:fructose (1.8 g/min at 14.4%) ingested during the cycle section of a simulated OD triathlon significantly improved subsequent 10km run performance by 4.0% compared to placebo, with no additional GI distress. First study to show significant triathlon performance improvement with CHO ingestion.
Bonacci et al.	2013	[60]	Controlled bike-to-run (laboratory)	Cross-sectional	18 (13M/5F)	Moderately-trained triathletes (≈4yrs experience)	Not reported	4	Triathletes whose running economy improved post-cycling had lower average cycling RPE than those whose RE was unchanged, while those whose RE was impaired had higher RPE in the final cycling minute. The findings suggest that perceived effort during cycling—particularly end-of-cycle exertion—is associated with subsequent running economy response.
Etxebarria et al.	2013	[61]	Controlled bike-to-run (field)	Crossover (3 conditions)	12 (12M)	Trained triathletes (8hr cycling + 3 hr running weekly)	Not reported	2, 4	A positive correlation was found between increased blood lactate and RPE during cycling and decrements in subsequent 9.3 km running performance. Variable-power cycling elicited higher [La], higher RPE at 60 min, and higher HR at 40 and 60 min compared to constant-power cycling, indicating cycling strategy modulates physiological/perceptual responses that predict transition running outcomes.
Rendos et al.	2013	[62]	Controlled bike-to-run (laboratory)	Single-group repeated measures	28 (13M/16F)	Triathletes (>2 yrs experience)	Not reported	3	During the first 14 min of post-cycling running, triathletes showed increased hip flexion, increased anterior pelvic tilt, and increased spine extension compared to control runs, with greater magnitude of changes in less experienced triathletes. The findings indicate that triathlon experience level influences trunk and pelvic kinematic adjustments after cycling.
Viker and Richardson	2013	[63]	Controlled bike-to-run (field)	Randomised crossover (non-blind)	13 triathletes	Well-trained triathletes	Not reported	2, 4	In a within-subjects randomised controlled trial comparing aft-placed versus traditionally placed shoe cleats during a 30-min simulated draft-legal triathlon cycling leg followed by a maximal 5-km run, total 5-km run times were similar between conditions (1084 ± 80 s vs. 1072 ± 64 s for aft and traditional, respectively). Running speed during the first kilometre was 2.1% ± 1.8 faster ($p < 0.05$) with the traditional cleat placement, while contact time, heart rate, and running speed for the remaining kilometres did not differ between cleat positions; cardiovascular measures during cycling also did not differ. The authors concluded that an aft

Author	Year	Ref.	Study Type	Design	Sample	Level	Age	Sub-RQs	Main Finding
									cleat position provides no beneficial effects on subsequent running performance in short-distance triathlon.
Etxebarria et al.	2014	[64]	Controlled bike-to-run (laboratory)	Counterbalanced crossover	9 (9M)	Well-trained triathletes (>3 yrs experience)	Not reported	2, 4	Both constant-PO and variable-PO cycling resulted in elevated VE, RPE, [La], and (in VAR only) HR during subsequent running compared to a control run, with higher running velocity at 4 mM blood lactate in the control condition. The findings demonstrate that isolated running does not directly replicate physiological responses of running after cycling, regardless of cycling power strategy.
Connick and Li	2015	[22]	Controlled bike-to-run (laboratory)	Repeated measures	8 (sex not specified; anthropometrics suggest all male)	Trained recreational (4+ yrs experience, IRONMAN-oriented)	41.9±6.0	3, 4	After 3h cycling, triathletes showed a large increase in stride time variability (+45%, d=0.95) and shorter strides at transition run onset, indicating disrupted locomotor control. Changes were transient — ankle and hip ROM recovered by 10 min of running. STV proposed as novel non-invasive measure of bike-to-run transition quality.
Johnson et al.	2015	[12]	Race-data observational (IRONMAN)	Cross-sectional	40 (29M, 11F)	Age-group qualifiers for IRONMAN World Championship (highly trained)	Men 45±11 y, Women 43±11 y	1, 2, 4, 5	In age-group athletes at the 2012 IRONMAN World Championship in Kona, those who maintained a speed closer to their goal pace on the downhill segments of both the bike and run achieved relatively better performance. High performers showed smaller heart-rate fluctuations between uphill and downhill sections (i.e., they maintained a more constant intensity), whereas low performers tended to reduce their intensity substantially on the downhill sections. These findings suggest that relative success depends on setting a realistic goal pace combined with maintaining a consistent intensity across changes in elevation.
Walsh et al.	2015	[65]	Controlled bike-to-run (laboratory)	Single-group repeated measures	6 triathletes	Trained triathletes (≈4.4 yrs experience)	Not reported	3, 4	No significant changes were observed in muscle EMG patterns between control and transition runs. The findings indicate that under moderate cycling intensity with variable cadence, prior cycling did not detectably alter neuromuscular activation patterns during subsequent running.
Angehrn et al.	2016	[11]	Race-data observational (IRONMAN)	Cross-sectional	1,392 (1,263M, 129F)	Top 100 finishers (elite + fast age-group)	Not reported	1, 2, 5	Analyzing split times of the top 100 finishers across 2014 IRONMAN races (13 cycling, 11 running), elite triathletes adopted positive pacing, slowing over time in 10 of 13 cycling races and all 11 running races. Women were significantly slower than men in cycling in 6 of 13 races, but no sex differences were found in running pacing. Women used the same positive pacing strategy as men.
Pageaux et al.	2017	[66]	Controlled bike-to-run (laboratory)	Randomised crossover (3 conditions)	9 (9M)	Trained triathletes (MAS 19.5 km·h^{-1})	Not reported	2, 4	After 1 hour of cycling, knee extensor MVC torque, twitch torque, doublet torque, and twitch rate of force development all decreased, with increased post-cycling RPE. Compared to matched-HR uphill walking, cycling induced a greater decrease in knee extensor force production capacity, suggesting cycling-specific neuromuscular fatigue mechanisms beyond cardiovascular load.

Author	Year	Ref.	Study Type	Design	Sample	Level	Age	Sub-RQs	Main Finding
Walsh et al.	2017	[67]	Controlled bike-to-run (laboratory)	Single-group pre-post	8 (8M)	Trained triathletes (≈4.8 yrs experience)	Not reported	3, 4	Post-cycling running showed increased stride frequency, decreased stride length, and increased cost of running compared to control runs, with elevated VE/VCO_2 and breathing frequency, decreased RER, and decreased tidal volume. The findings indicate that prior moderate-intensity variable-cadence cycling significantly affects both spatiotemporal running parameters and cardiorespiratory responses during subsequent running.
Käch et al.	2018	[14]	Race-data observational (IRONMAN)	Cross-sectional	410,881 (329,066M, 81,815F) — all finishers after exclusion	Pro + age group (18-24 to 75-79); all finishers = recreational to elite range	5-year age groups: 18-24, 25-29, 30-34, …, 75-79	5	An analysis of all IRONMAN finishers worldwide—covering 253 races and approximately 410,000 finishers—showed that age-related performance decline began at 25–29 years for swimming, and at 30–34 years for women and 35–39 years for men in both cycling and running. Analyzing all finishers revealed an earlier age of onset of decline than studies limited to the top 10, demonstrating that the decline patterns of recreational athletes differ from those of elite athletes. In running, a polarized trend was observed, with younger age groups improving while middle-aged and older groups declined.
Pryor et al.	2018	[13]	Race-data observational (IRONMAN)	Case study	1 (0M, 1F)	Overall female winner("Alice", IM PB 9:20)	26 years	1, 2, 4	The female overall winner of IRONMAN Lake Placid adopted an oscillatory pacing strategy in response to the large elevation changes, allowing velocity to fluctuate while keeping heart rate and power output constant. The variation in physiological load was very small—a bike normalized power (NP) MAPE of 2.7% and a run heart-rate MAPE of 1.2%—demonstrating faithful execution of a pre-planned pacing strategy.
Olcina et al.	2019	[17]	Controlled bike-to-run (field)	Crossover	10 (8M/2F)	Trained (moderate level, regional/national)	25.7±8.9	2, 3, 4	Prior cycling impaired 12-min running performance by ~6%, with decreased stride length and elevated muscle O2 saturation (indicating inability to peripherally utilise oxygen). SmO2 via wearable NIRS showed largest effect size, suggesting it as a sensitive real-time monitoring tool for brick training.
Weich et al.	2019	[68]	Controlled bike-to-run (field)	Repeated measures (2 conditions)	36 (24M/12F)	Trained endurance athletes (≈10 hr/wk)	Not reported	3	No main effects of time or interactions with transition run were observed in 3D ankle acceleration patterns; both isolated and transition runs showed similar pattern changes over time. The findings suggest that, at threshold-based moderate cycling intensity, post-cycling running does not differentially alter ankle-level acceleration patterns compared to isolated running.
da Rosa et al.	2019	[21]	Controlled bike-to-run (laboratory)	Single-group pre-post	14 (sex not specified; anthropometrics suggest predominantly male)	Middle-level (regional)	30±5	3, 4	Prior cycling increased running CoT by ~12% in middle-level triathletes, but mechanical efficiency was preserved through compensatory stride adjustments (shorter stride length, higher frequency). Kleg and Kvert decreased only at the end of 20-min post-cycling run, indicating progressive spring-mass system compliance.
du Plessis et al.	2020	[69]	Controlled bike-to-run (laboratory)	Single-group pre-post	17 (17M)	Trained triathletes (4 yrs experience)	Not reported	4	Running after 60 min of cycling elicited elevated VO_2, oxygen cost, aerobic energy cost, VE, HR, RPE, and percentage of maximum effort, with decreased RER, compared to a control run. The findings establish that prior cycling robustly

Author	Year	Ref.	Study Type	Design	Sample	Level	Age	Sub-RQs	Main Finding
									increases physiological cost and perceptual responses of subsequent running, regardless of how running economy is calculated.
Evans et al.	2021	[70]	Controlled bike-to-run (field)	Non-randomised repeated measures	7 (sex not specified)	Recreational	40±10 yrs; 173±8.9cm; 71±8.1kg	3, 4	Posterior cleat cycling significantly reduced trunk acceleration magnitudes in subsequent 5km running ($p<0.0001$, d=0.9) with lower RPE (12.71→11.85, $p=0.02$) and faster run time (22.6→22.1 min), despite increasing trunk acceleration during cycling itself. Wearable IMU successfully detected cleat-dependent kinematic changes in outdoor setting.
Fraeulin et al.	2021	[71]	Controlled bike-to-run (field)	Crossover	16 M (22 recruited, 6 dropped due to data quality)	Elite long-distance (targeting sub-9:30 IRONMAN)	31.3±5.4 (reported as 32.1±6.0 in methods)	3, 5	Transition running showed complex kinematic differences from isolated running in overground conditions: pronounced trunk extension, increased hip flexion in swing, altered knee flexion patterns. These occurred despite no changes in step length or rate, suggesting the 'brick running' incoordination involves joint-level kinematics rather than spatiotemporal parameters.
Jacques et al.	2021	[72]	Controlled bike-to-run (laboratory)	Repeated measures (bilateral comparison)	13 M (11 for EMG analysis; 2 excluded due to noise)	Competitive triathletes, injury-free (no lower limb injury in prior 6 months)	34±5.7	3, 4, 5	Running after cycling induces inter-limb differences in triceps surae muscle activation (SOL: moderate effect at End stage) but not in kinetics, kinematics, or tendon strain. SPM time-series analysis revealed differences that 0d metrics would have missed. Inter-limb SOL asymmetry during push-off may represent a potential overuse injury mechanism in triathletes. Running speed increased from Start to Mid/End ($P=0.03$, ES~0.94).
Evans et al.	2022	[73]	Controlled bike-to-run (field)	Randomised repeated measures	10 (7M/3F)	Competitive recreational	32±3.2 yrs; 71.4±2.2 kg	3, 4	Cycling in aero position significantly reduced LN and ML CoM acceleration compared to drops, despite higher RPE. Subsequent 5km running after aero cycling showed significantly less LN and ML acceleration with lower RPE (10.6 vs 11.5) and faster run time (22:56 vs 23:34, $p<0.001$). Triathletes appeared to self-regulate CoM trajectory during initial 1km of post-aero running.
Mateus et al.	2022	[74]	Controlled bike-to-run (laboratory)	Repeated measures	20 (20M / 0F)	Well-trained triathletes preparing for IRONMAN (3 months out)	33.2±6.6 yrs	3, 4	Cycling RTE increased HR and RPE in subsequent running but did not alter plantar pressure or load distribution. Contact time increased POST-RTE on level and uphill only. Incline was a stronger modifier of running mechanics than cycling-induced fatigue in well-trained triathletes.
Stewart et al.	2022	[75]	Controlled bike-to-run (laboratory)	Counterbalanced crossover	8 (7M/1F)	Competitive club-level collegiate triathletes	21.1±0.5	2, 4	Mechanical efficiency of running was significantly reduced after 40-km cycling TT compared to 5-km run TT in collegiate triathletes, but running economy (VO_2) was unchanged. Anaerobic energy expenditure was significantly elevated after cycling, indicating a metabolic shift rather than an aerobic cost increase.
Takahashi et al.	2022	[76]	Controlled bike-to-run (laboratory)	Cross-sectional (between-group)	18 (10 triathletes + 8 runners);	Trained (triathletes: 2.3±1.4 yrs competition;	Not reported in main text (see supplements)	3, 4	Participants with decreased SSC function after cycling (RSI -14.4%) showed increased VO_2 (+1.6%), VE (+4.4%), and HR (+5.3%) in transition run, while those without SSC decrease showed no VO_2/VE changes. SSC function recovered after 15 min running. Effects of cycling on subsequent running did not differ between triathletes and runners.

Author	Year	Ref.	Study Type	Design	Sample	Level	Age	Sub-RQs	Main Finding
					sex not specified	runners: 5.2±3.0 sessions/week)			
Takahashi et al.	2022	[77]	Controlled bike-to-run (laboratory)	Single-group pre-post	11 M	Highly trained university triathletes	22.4±3.5	4, 5	First study to demonstrate that SSC function decreases after cycling (RSI -10.7%, d=1.10). Individual variation was large. Brick training experience showed a large but non-significant correlation (r=0.62) with smaller SSC decreases, suggesting brick training may help preserve SSC function through the transition.
Walsh et al.	2022	[78]	Controlled bike-to-run (laboratory)	Repeated measures (multiple timepoints)	9 (5M/4F)	Highly trained / elite (National & World Triathlon level)	24.3±7.6	3	Prior moderate-intensity variable-cadence cycling significantly altered intersegmental coordination (run loop planarity) in elite triathletes during acute transition phase, but alterations were corrected by the 3rd minute of running (TRSS). PCA-based planar covariation analysis detected coordination changes not apparent in standard joint angle or stride rate measures.
Weich et al.	2022	[79]	Controlled bike-to-run (field)	Crossover	22 (10F/12M)	Well-trained (non-elite)	29±7.8 (F: 31±9.2, M: 28±6.1)	3, 4	The T2 'sluggishness' in triathlon running is linked to increased movement variability (δD) rather than changes in running pattern (δM) itself. The transient effect lasted longest after cycling (~679 m) vs matched running preload (~450 m) vs isolated run (~294 m), confirming cycling specifically disrupts running precision. Attractor Method detected differences invisible to conventional stride analysis.
Knechtle et al.	2023	[10]	Race-data observational (IRONMAN)	Cross-sectional	60 (30M, 30F)	Professional (Top 30 overall finishers, pro category)	Not reported	1, 2, 5	Among the top 30 professionals at IRONMAN Hawaii 2022, men adopted negative pacing in cycling, while women adopted even pacing. In the run, the top 10 men and all the women's groups showed slightly positive pacing, whereas men placing 11th–30th showed more pronounced positive pacing (a decline in speed); groups with lower running pacing variability tended to place higher.
Vivan et al.	2024	[31]	Controlled bike-to-run (laboratory)	Randomised crossover	38 (21M / 17F)	Well-trained amateur triathletes	M: 32.1±8.3, F: 40.2±8.1 yrs	2, 4, 5	Well-trained amateur triathletes cycling 20km at varied FTP% then running 5km TT: males ran significantly slower after 90% vs 80% FTP (~35s), but C+R time was fastest at 90% FTP for both sexes. Females showed no significant running impairment across intensities.
Dos Anjos et al.	2025	[80]	Controlled bike-to-run (field)	Cross-sectional (between-sex)	32 (16M / 16F)	Amateur triathletes	M: 34.7±7.5, F: 39.5±7.7 yrs	2, 5	After 20km cycling at 90% FTP, males ran 5km ~11% faster than females. Pacing strategy differed: males performed an end-sprint in the final 600m, whereas females maintained constant pace throughout.
Röhrs et al.	2025	[81]	Controlled bike-to-run (laboratory)	Repeated measures	19 (4F/15M)	Well-trained junior (national squad)	17.2±1.8 (range 14-21)	2, 4, 5	Despite trivial/small group-level changes in OC/EC after strenuous cycling, a pronounced CHO→FO substrate shift occurred in junior squad triathletes. This shift, combined with VO2peak and OC, predicted 88% of fatigued running performance variance, supporting substrate utilization as a durability mechanism in youth triathletes.

Sub-RQ definitions: 1 (Where) — the extent to which running performance decline has been studied at IRONMAN race distance, as opposed to shorter races and controlled protocols; 2 (How much) — the extent to which the magnitude of running performance decline has been quantified, both within-race and relative to standalone running ability; 3 (How) — physiological and biomechanical changes observed during the bike-to-run transition and within the run segment; 4 (Why) — causal mechanisms proposed to explain running performance decline; 5 (Who) — factors contributing to inter-individual differences in the magnitude of decline.

Supplementary Table S2. Complete search strings by database (searched February 25, 2026).

Database	Step	Search String	Records
PubMed (NCBI)	Single query	((ironman[tiab] OR "iron man"[tiab] OR "iron-distance"[tiab] OR "long-distance triathlon"[tiab] OR "full-distance triathlon"[tiab] OR "ultra-distance triathlon"[tiab]) AND (run[tiab] OR running[tiab] OR marathon[tiab] OR pacing[tiab] OR "run split"[tiab] OR "running performance"[tiab] OR "race time"[tiab] OR "finish time"[tiab])) OR ((triathlete*[tiab] OR triathlon[tiab]) AND (cycling[tiab] OR cycle[tiab] OR bike[tiab] OR bicycle[tiab]) AND (run[tiab] OR running[tiab] OR "running performance"[tiab] OR stride[tiab] OR "transition run"[tiab] OR "run performance"[tiab]))	681
SPORTDiscus (EBSCOhost)	S1: Block 1 — IRONMAN distance	TI(ironman OR "iron man" OR "long-distance triathlon" OR "full-distance triathlon" OR "ultra-distance triathlon") OR AB(ironman OR "iron man" OR "long-distance triathlon" OR "full-distance triathlon" OR "ultra-distance triathlon")	3,950
	S2: Block 1 — Run terms	TI(run OR running OR marathon OR pacing OR "run split" OR "running performance" OR "race time" OR "finish time") OR AB(run OR running OR marathon OR pacing OR "run split" OR "running performance" OR "race time" OR "finish time")	112,969
	S3: Block 1 combined	S1 AND S2	822
	S4: Block 2 — Triathlon	TI(triathlete* OR triathlon) OR AB(triathlete* OR triathlon)	12,681
	S5: Block 2 — Cycling	TI(cycling OR cycle OR bike OR bicycle) OR AB(cycling OR cycle OR bike OR bicycle)	95,247
	S6: Block 2 — Run terms	TI(run OR running OR "running performance" OR stride OR "transition run" OR "run performance") OR AB(run OR running OR "running performance" OR stride OR "transition run" OR "run performance")	97,862
	S7: Block 2 combined	S4 AND S5 AND S6	1,163
	S8: Final combined	S3 OR S7	1,724
	S9: Filtered	S8 limited to Source Type: Academic Journals	600
Combined		PubMed + SPORTDiscus (S9), deduplicated	896

Supplementary Table S3. Studies excluded at full-text screening (n = 11), with the reason for each exclusion.

Excluded study	Reason for exclusion	Full publication superseding the excluded abstract
Bonacci, J.; Chapman, A.; Blanch, P.; Vicenzino, B. The direct effect of cycling on neuromuscular control during running in lesser trained triathletes. J. Sci. Med. Sport 2010, 12, e50–e51. https://doi.org/10.1016/j.jsams.2009.10.106	Conference abstract superseded by a full peer-reviewed publication	Bonacci, J.; Blanch, P.; Chapman, A.R.; Vicenzino, B. J. Sports Sci. 2010, 28, 1477–1487 (included; cited as reference 25 in the main text)
Bonacci, J.; Saunders, P.; Alexander, M.; Blanch, P.; Chapman, A.; Vicenzino, B. Run performance and neuromuscular control is not affected by cycling in elite international triathletes. J. Sci. Med. Sport 2010, 13S, e26–e27. https://doi.org/10.1016/j.jsams.2010.10.517	Conference abstract superseded by a full peer-reviewed publication	Bonacci, J.; Saunders, P.U.; Alexander, M.; Blanch, P.; Vicenzino, B. Sports Biomech. 2011, 10, 59–71 (included; cited as reference 24 in the main text)
Bonacci, J.; Green, D.; Saunders, P.; Franettovich, M.; Chapman, A.; Blanch, P.; Vicenzino, B. Is plyometric training effective for correcting altered running neuromotor control post-cycling in triathletes? J. Sci. Med. Sport 2010, 13S, e81. https://doi.org/10.1016/j.jsams.2010.10.633	Conference abstract superseded by a full peer-reviewed publication	Bonacci, J.; Green, D.; Saunders, P.U.; Franettovich, M.; Blanch, P.; Vicenzino, B. Phys. Ther. Sport 2011, 12, 15–21 (included; cited as reference 56 in the main text)
Chapman, A.; Vicenzino, B.; Hodges, R.; Dowlan, S.; Hahn, A.; Alexander, M.; Milner, T. Cycling impairs neuromuscular control during running in triathletes: implications for performance, injury and intervention. J. Sci. Med. Sport 2009, 12, S61. https://doi.org/10.1016/j.jsams.2008.12.143	Conference abstract superseded by a full peer-reviewed publication	Full peer-reviewed publications by the same group are included in the review; the specific publication corresponding to this abstract could not be established
McGawley, K.; Shannon, O. The effect of carbohydrate feeding during cycling on run performance within a simulated Olympic-distance triathlon. J. Sci. Med. Sport 2010, 13S, e38. https://doi.org/10.1016/j.jsams.2010.10.543	Conference abstract superseded by a full peer-reviewed publication	McGawley, K.; Shannon, O.; Betts, J. Appl. Physiol. Nutr. Metab. 2012, 37, 664–671 (included; cited as reference 27 in the main text)
Jones, M. An analysis of the biomechanics of triathletes during the bike-run transition and run. Arch. Phys. Med. Rehabil. 2019, 100, e43.	Full text could not be obtained	—
Kreider, R.B. Effects of cycling on running performance in triathletes. Ann. Sports Med. 1988, 3, 220–225.	Full text could not be obtained	—
Lepers, R.; Knechtle, B.; Stapley, P.J. Trends in triathlon performance: effects of sex and age. Sports Med. 2013, 43, 851–863. https://doi.org/10.1007/s40279-013-0067-4	Review article with no original data	Lepers, R.; Knechtle, B.; Stapley, P.J. Sports Med. 2013, 43, 851–863 (not included; cited as reference 3 for context)
Walsh, J.A. The rise of elite short-course triathlon re-emphasises the necessity to transition efficiently from cycling to running. Sports 2019, 7, 99. https://doi.org/10.3390/sports7050099	Review article with no original data	—
Suriano, R.; Bishop, D. Effects of cycle strategy and fibre composition on muscle glycogen depletion pattern and subsequent running economy. Br. J. Sports Med. 2010, 44, 443–448. https://doi.org/10.1136/bjsm.2007.046029	Primary outcome was glycogen depletion and running economy at a fixed running speed; run performance decline was not measured	—
Etxebarria, N.; Anson, J.M.; Pyne, D.B.; Ferguson, R.A. High-intensity cycle interval training improves cycling and running performance in triathletes. Eur. J. Sport Sci. 2014, 14, 521–529.	Training intervention study without quantitative measurement of run performance decline following a cycling segment	—

Reference numbers refer to the reference list of the main manuscript. The three excluded studies marked as superseded by an identified publication were matched to that publication on the basis of the study design, the number of participants, and the authorship.
